# The Neuromuscular Origins of Kinematic Variability during Perturbed Walking

**DOI:** 10.1038/s41598-017-00942-x

**Published:** 2017-04-11

**Authors:** Heather E. Stokes, Jessica D. Thompson, Jason R. Franz

**Affiliations:** Joint Department of Biomedical Engineering, University of North Carolina at Chapel Hill and North Carolina State University, Chapel Hill, NC USA

## Abstract

We investigated the neuromuscular contributions to kinematic variability and thus step to step adjustments in posture and foot placement across a range of walking speeds in response to optical flow perturbations of different amplitudes using a custom virtual environment. We found that perturbations significantly increased step width, decreased step length, and elicited larger trunk sway compared to normal walking. However, perturbation-induced effects on the corresponding variabilities of these measurements were much more profound. Consistent with our hypotheses, we found that: (1) perturbations increased EMG activity of the gluteus medius and postural control muscles during leg swing, and increased antagonist leg muscle coactivation during limb loading in early stance, and (2) changes in the magnitude of step to step adjustments in postural sway and lateral foot placement positively correlated with those of postural control and gluteus medius muscle activities, respectively, in response to perturbations. However, (3) interactions between walking speed and susceptibility to perturbations, when present, were more complex than anticipated. Our study provides important mechanistic neuromuscular insight into walking balance control and important reference values for the emergence of balance impairment.

## Introduction

Walking balance control involves coordinated adjustments in posture (i.e., head and trunk stabilization) and foot placement from step to step^1–4^. Particularly in unpredictable and challenging environmental conditions, these adjustments depend on the integration of reliable sensory feedback and the planning and execution of appropriate motor responses^5, 6^. Accordingly, sensory and mechanical perturbations are increasingly used to study corrective motor responses in walking as well as the onset and progression of balance deficits^4, 6–12^. Kinematic variability is frequently used to quantify the magnitude and efficacy of balance corrections in the presence of perturbations^6, 8–10^. However, few studies have investigated the neuromuscular mechanisms underlying kinematic variability and thus step to step adjustments in posture and foot placement during walking. This is especially notable for responses to visual (i.e., optical flow) perturbations^6, 8–11^, which elicit profound increases in kinematic variability only through the visual perception of imbalance.

Electromyographic (EMG) recordings of muscle activity during perturbed walking can provide insight into the extent to which dynamic balance corrections are neurally mediated via muscular actions. For example, by imposing lateral mechanical perturbations of the swing leg, Rankin *et al*.^4^ revealed the important role of swing phase gluteus medius activity in adjusting foot placement laterally with each step, thereby regulating mediolateral translation of the trunk relative to the stance foot^4^. However, the neuromuscular adjustments underlying corrections in response to optical flow perturbations during walking may have unique biomechanical origins. Specifically, we recently proposed that optical flow perturbations elicit postural disturbances in walking via visuomotor entrainment, wherein head and trunk kinematics synchronize to the perturbation via postural adjustments to instinctively unify visual with somatosensory and vestibular feedback^10^. We suspect that these postural disturbances subsequently elicit step to step adjustments in foot placement, particularly in the mediolateral direction, to preserve whole-body balance. Thus, our observations allude to potentially coordinated changes in postural control muscle activities to orchestrate postural adjustments with those of gluteus medius activity to regulate lateral foot placement from step to step. However, kinematic measurements alone are unable to discern passive mechanics from active control in these postural and foot placement adjustments. Indeed, we also observe robust entrainment of foot trajectories to even the most complex optical flow perturbations^10^, which is unlikely to be explained by leg muscle adjustments alone.

In addition to the step to step kinematic adjustments used to accommodate balance perturbations during walking, which we suspect occur predominantly during leg swing, those perturbations may also require increased leg joint stability, particularly during limb loading in early stance. The concurrent activation of antagonist muscles is a well-documented phenomenon in motor control thought to increase joint stiffness to improve joint stability, thereby mitigating the effects of unexpected environmental demands or perturbations^13–15^. For example, we and others have shown that older adults walk with greater lower leg (i.e., ankle flexor-extensor) and upper leg (i.e., knee flexor-extensor) antagonist muscle coactivation than young adults^13, 15, 16^. Moreover, at least in arm reaching tasks, antagonist muscle coactivation and limb stiffness increase in the presence of external force perturbations^14^. However, the neuromuscular response of antagonist leg muscles, and thus leg joint stability, when accommodating sensory perturbations during walking remains unclear.

Finally, walking speed may also systematically influence the relation between kinematic variability and muscle activity in the presence of balance perturbations. During normal, unperturbed walking, Dingwell and Marin^17^ simultaneously analyzed dynamic stability and kinematic variability and suggested that walking slower improves one’s resilience to perturbations, despite increasing their movement variability. However, dynamic stability quantifies resilience to small perturbations arising normally during walking, and may not reflect the response to larger balance disturbances. Intuitively, compared to walking faster, walking slower would provide more time to plan and execute appropriate kinematic adjustments to maintain balance in the presence of perturbations. Accordingly, balance perturbations should elicit smaller increases in kinematic variability and muscle activity when walking at slower speeds. However, Goodworth *et al*.^7^ recently found that postural sway was less sensitive to surface perturbations when applied at a speed modestly faster than preferred^7^. Thus, this premise remains equivocal.

Therefore, the purpose of this study was to investigate the association between step to step kinematic adjustments and specific changes in muscle activity across a range of walking speeds using optical flow perturbations of different amplitudes. First, we hypothesized that perturbations would increase EMG activity of the gluteus medius and postural control muscles during leg swing, and increase the coactivation of antagonistic leg muscles during limb loading in early stance. Second, we hypothesized that changes in the magnitude of step to step adjustments in postural sway and lateral foot placement would be positively correlated with those of postural control and gluteus medius muscle activities, respectively, in response to perturbations. Finally, we hypothesized that perturbation-induced changes in kinematic variability and EMG activity would be smaller at slower walking speeds.

## Methods

### Subjects and Experimental Protocol

Twelve healthy young adults (7 females, mean ± standard deviation, age: 25 ± 4.6 yrs, height: 1.70 ± 0.10 m, mass: 65.3 ± 10.8 kg) participated in this study. The experimental protocol was approved by and performed in accordance with the relevant guidelines and regulations of the University of North Carolina Internal Review Board, and subjects provided written informed consent prior to participating. Subjects also completed a health questionnaire prior to participating to ensure they had no known neuromuscular, cardiovascular, or orthopedic diseases.

**Figure 1:**
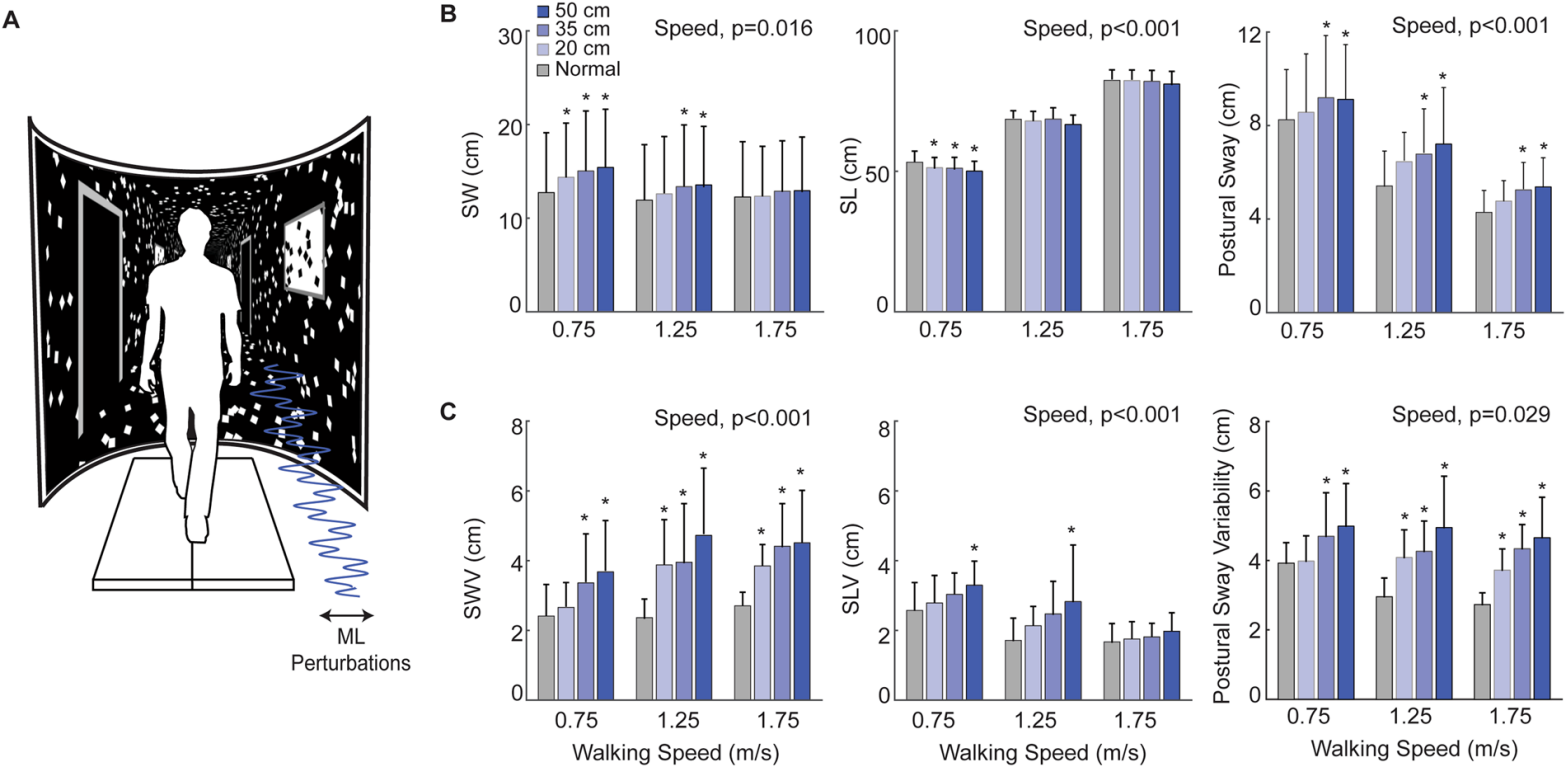
(**A**) Subjects walked in an immersive virtual environment while watching a hallway with and without continuous mediolateral optical flow perturbations. Group average (±standard deviation) foot placement and trunk kinematic (**B**) magnitudes and (**C**) variabilities across the three walking speeds during normal and visually perturbed walking. Asterisks (*) indicate significantly different from normal, unperturbed walking in Tukey’s post-hoc pairwise comparisons (p < 0.05). SW: Step width; SL: Step length; SWV: Step width variability; SLV: Step length variability.

All testing was completed on a dual-belt, force-measuring treadmill (Bertec, Inc., Columbus, OH) surrounded by an immersive, semi-circular curved screen measuring 2.24 m height and 2.83 m wide (Fig. 1A). Subjects started by walking on the treadmill at a comfortable speed for 5 minutes to acclimate to the walking environment and allow their movement patterns to stabilize. Subjects were then fit to an overhead harness which they wore for the duration of the experiment. In fully randomized order, subjects then completed a series of twelve 2-minute walking trials while watching a speed-matched virtual hallway projected onto the curved screen. During some trials, motion of the virtual hallway included continuous mediolateral (ML) optical flow perturbations consisting of a sum of three sinusoids, such that the full amplitude was applied at 0.250 Hz and half that amplitude was applied at 0.125 Hz and 0.442 Hz. The perturbations were consistent with the visual feedback associated with head movements, such that the hallway’s end moved very little compared to that in the foreground, thereby inducing balance corrections rather than heading corrections. At each of three speeds (0.75, 1.25, and 1.75 m/s), subjects walked both normally (i.e., unperturbed) and with each of three perturbation amplitudes (20, 35, and 50 cm). We opted to analyze the measurements outlined below during the second minute of each two-minute trial to allow some time for subjects to reach a steady state neuromuscular response to the ongoing perturbations. Finally, we used a photocell timing system (Brower Timing, Draper, UT) to measure subjects’ preferred overground walking speed from the average of three times taken to traverse the middle 2 m of a 10 m walkway at their normal, comfortable speed (i.e., 1.29 ± 0.18 m·s^−1^).

### Measurements and Data Analysis

A 14-camera motion capture system (Motion Analysis, CA) tracked 30 retro-reflective fmarkers placed on the subject’s pelvis, right and left legs, and cervical spine (C7). Three-dimensional marker trajectories were collected at 100 Hz and then low-pass filtered using a 4th order Butterworth filter and a cut-off frequency of 8 Hz. Heel strikes were identified from peaks in the fore-aft position of the heel markers relative to the sacral marker using published procedures^18^. From this data, we computed step widths (SW) from consecutive mediolateral heel positions which were averaged over a period from 12–25% of each stride corresponding to mid-stance prior to heel rise^19^. Step lengths (SL) were calculated as the relative anterior-posterior positions of consecutive heel markers at 20% of each stride plus the treadmill belt translation during that step^8^. Using time series of SW and SL, we computed their respective variabilities (SWV and SLV) as the standard deviation over all steps taken during each trial. Finally, from the kinematic data, we quantified postural sway as the average and standard deviation of peak-to-peak mediolateral (ML) trunk motion, defined using the trajectory of a marker placed over the C7 vertebra.

In addition, after preparing the shaved skin with alcohol, we collected electromyographic (EMG) recordings of right leg and postural control muscles using wireless single differential electrodes (Trigno, Delsys, Inc., Boston, MA). Following the guidelines of Cram and Kasman^20^, we collected muscle activities at 1000 Hz of the following ten muscles: medial gastrocnemius (MG), soleus (SOL), tibialis anterior (TA), peroneous longus (PL), vastus lateralis (VL), medial hamstring (MH), gluteus medius (GM), external oblique (EO), and erector spinae (ES). We visually inspected the EMG signals while subjects contracted each instrumented muscle to ensure signal quality and electrode placement. A custom MATLAB script processed all of the EMG data (Mathworks, Inc., Natick, MA). EMG activity, bandpass filtered (20–450 Hz), rectified, and normalized to the mean value during unperturbed walking at 1.25 m/s, was averaged within six gait cycle phases: early (0–10%), midstance (10–30%), terminal stance (30–60%), initial swing (60–73%), midswing (73–87%), and terminal swing (87–100%)^19^.

### Statistical Analysis

We calculated mean values of step kinematics, postural sway variability, and EMG amplitudes across all strides for each subject. First, a two-way repeated measures analysis of variance (rmANOVA) tested for main effects of and interactions between walking speed (3 levels: 0.75, 1.25, and 1.75 m/s) and perturbation amplitude (4 levels: 0, 20, 35, and 50 cm). When a significant main effect or interaction was found, we performed post-hoc pairwise comparisons versus unperturbed walking using Tukey’s Honest Significant Difference to correct for multiple comparisons. We then used a series of correlations between specific changes in kinematic variability and those in EMG activity from unperturbed walking, pooled across all walking speeds and perturbation amplitudes, to test our third hypothesis. Specifically, we first calculated partial Pearson’s correlation coefficients between: (*i*) changes in step width variability and changes in GM activity, and (*ii*) changes in postural sway variability and changes in GM, EO, and ES activity, while controlling for walking speed as a covariate. We focused these correlation analyses to include GM, EO, and ES activity only within those gait cycle phases identified as having a significant main effect of perturbation amplitude in the rmANOVA. In our final planned comparison, for gait cycle phases identified as having concurrent significant main effects of perturbation amplitude among these three muscles, we calculated Pearson’s correlation coefficients between changes in GM activity and changes in EO and ES activity. For all comparisons, we defined significance using an alpha level of 0.05.

## Results

### Perturbation effects on kinematic variability and EMG activity

In response to perturbations, subjects took wider, shorter, and more variable steps and increased their postural sway variability compared to walking normally (perturbation main effect, p’s < 0.001) (Fig. 1B). For example, post-hoc comparisons revealed that, on average across the range of speeds tested, subjects took 14% wider (p < 0.001) and 3% shorter steps (p < 0.001), accompanied by 73% larger SWV (p < 0.001), 36% larger SLV (p < 0.001), and 52% larger postural sway variability (p < 0.001) in response to the largest amplitude perturbation (i.e., 50 cm).

**Figure 2:**
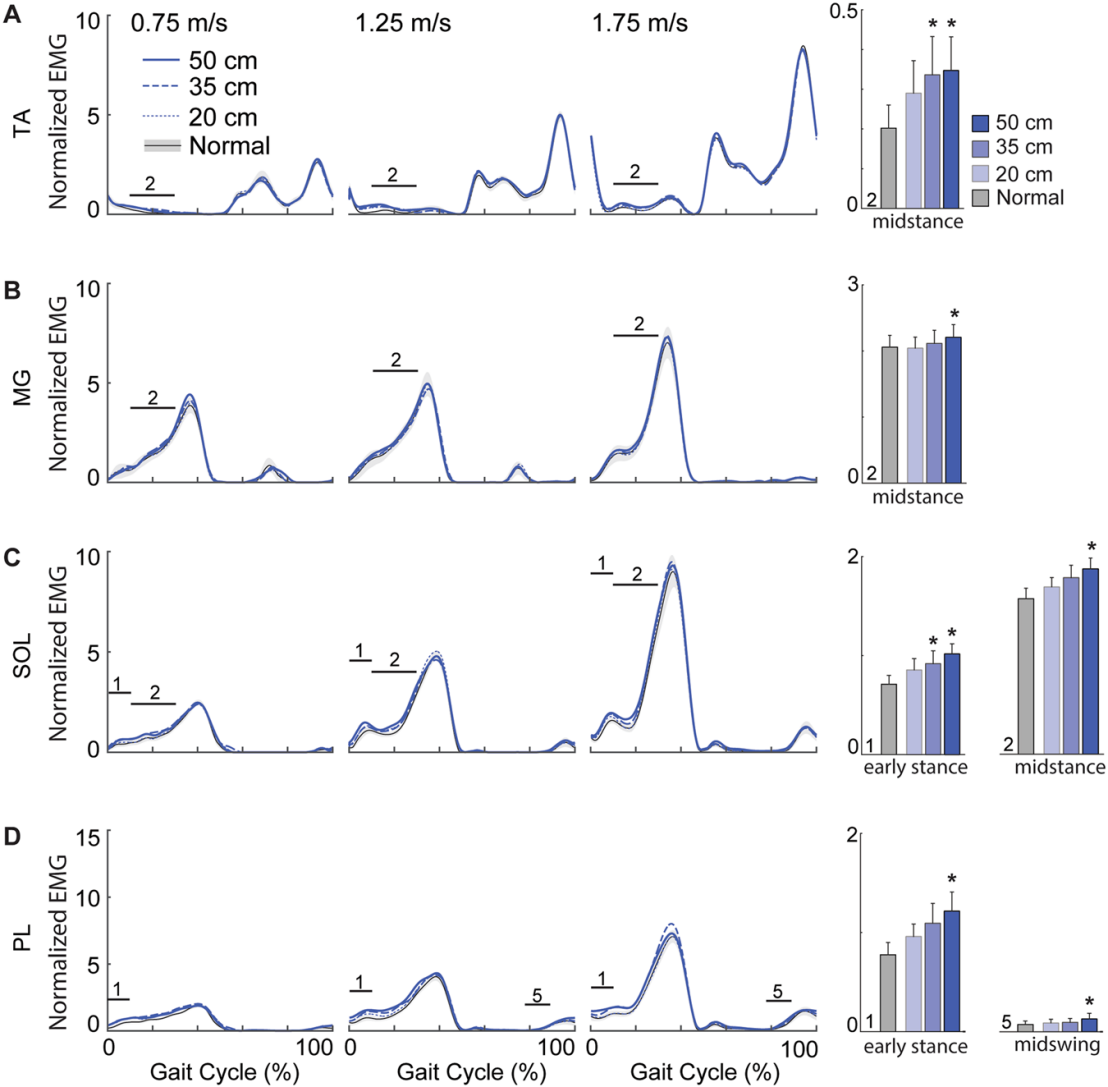
Perturbation-induced changes in lower leg muscle activities. Group average electromyographic (EMG) recordings from the lower leg muscles during normal (±standard error) and visually perturbed walking as a function of the gait cycle. Amplitudes are normalized to the mean values over a complete stride walking normally at 1.25 m/s. Solid horizontal lines with numerical indicators represent gait cycle phases with significant main effects of perturbation amplitude (p < 0.05) (i.e., 1: early stance; 2: midstance; 5: midswing). Each of these identified phases is also accompanied by bar graphs illustrating the group average (±standard error) muscle activity for each condition, pooled across walking speeds. Asterisks (*) indicate significantly different from normal, unperturbed walking in Tukey’s post-hoc pairwise comparisons (p < 0.05). (**A**) TA: Tibalis anterior; (**B**) MG: Medial gastrocnemius; (**C**) SOL: Soleus; (**D**) PL: Peroneus longus.

**Figure 3:**
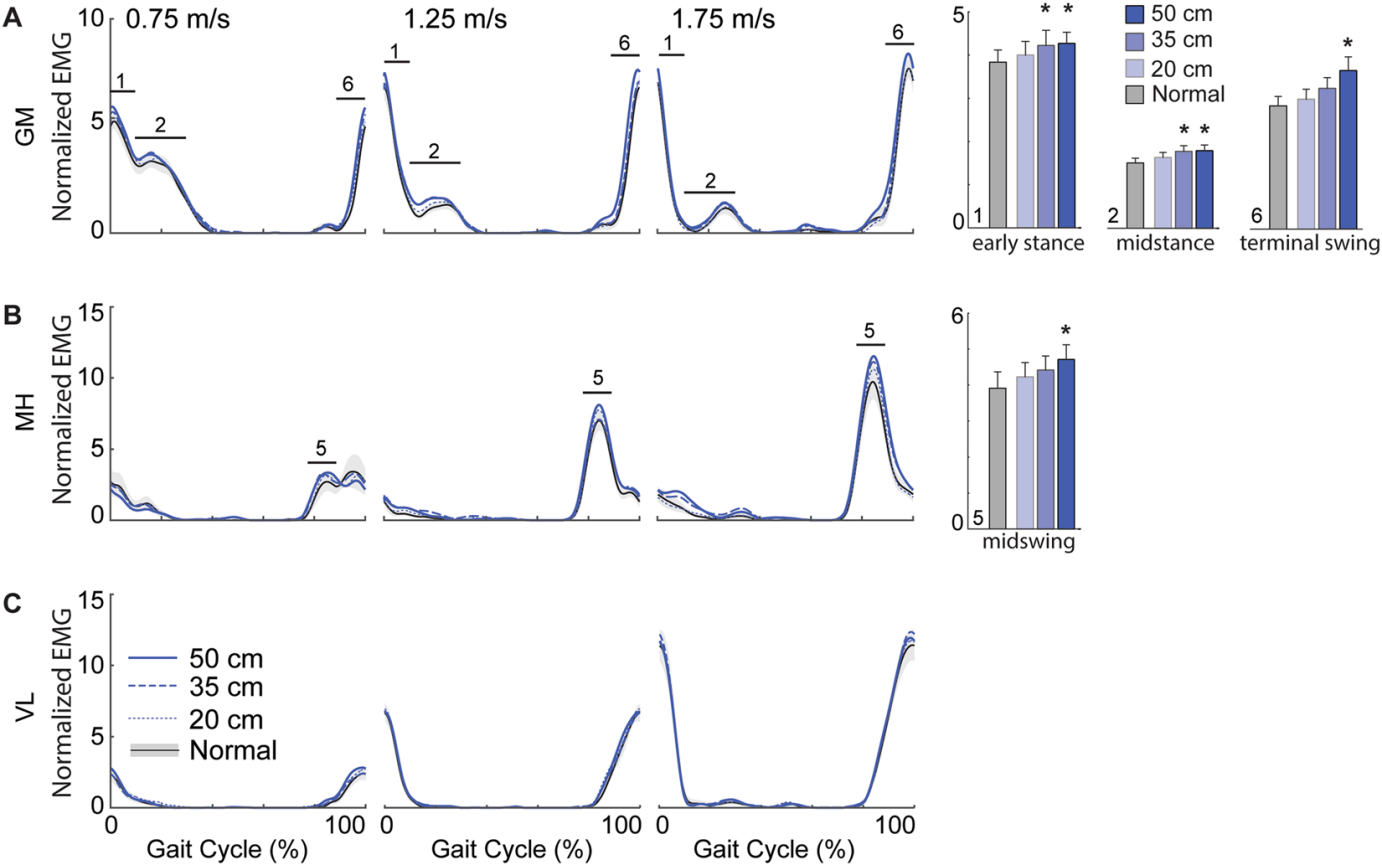
Perturbation-induced changes in upper leg muscle activities. Group average electromyographic (EMG) recordings from the thigh muscles during normal (±standard error) and visually perturbed walking as a function of the gait cycle. Amplitudes are normalized to the mean values over a complete stride walking normally at 1.25 m/s. Solid horizontal lines with numerical indicators represent gait cycle phases with significant main effects of perturbation amplitude (p < 0.05) (i.e., 1: early stance; 2: midstance; 5: midswing; 6: terminal swing). Each of these identified phases is also accompanied by bar graphs illustrating the group average (±standard error) muscle activity for each condition, pooled across walking speeds. Asterisks (*) indicate significantly different from normal, unperturbed walking in Tukey’s post-hoc pairwise comparisons (p < 0.05). (**A**) GM: Gluteus medius; (**B**) MH: Medial hamstring; (**C**) VL: vastus lateralis.

**Figure 4:**
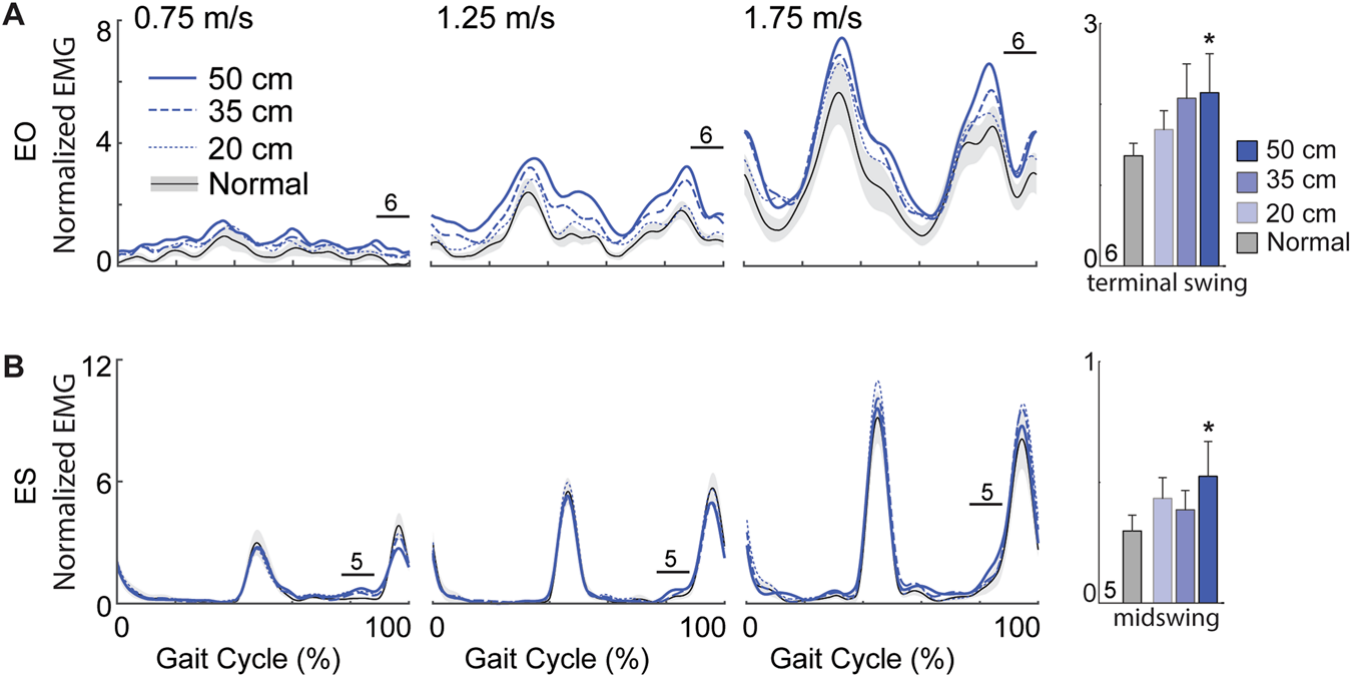
Perturbation-induced changes in postural control muscle activities. Group average electromyographic (EMG) recordings from postural control muscles during normal (±standard error) and visually perturbed walking as a function of the gait cycle. Amplitudes are normalized to the mean values over a complete stride walking normally at 1.25 m/s. Solid horizontal lines with numerical indicators represent gait cycle phases with significant main effects of perturbation amplitude (p < 0.05) (i.e., 5: midswing; 6: terminal swing). Each of these identified phases is also accompanied by bar graphs illustrating the group average (±standard error) muscle activity for each condition, pooled across walking speeds. Asterisks (*) indicate significantly different from normal, unperturbed walking in Tukey’s post-hoc pairwise comparisons (p < 0.05). (**A**) EO: External oblique; (**B**) ES: Erector spinae.

Accompanying these kinematic changes, EMG activity of all but one lower leg (Fig. 2), thigh (Fig. 3), and postural control muscle (Fig. 4) increased in the presence of optical flow perturbations. Moreover, these increases were phase-dependent. Lower leg muscles increased significantly during early to midstance in a manner consistent with larger antagonist coactivations, averaging a 72% increase in the TA (p = 0.026; Fig. 2A), a 19% increase in the MG and SOL (p’s < 0.025; Fig. 2B,C), and a 57% increase in the PL (p = 0.046; Fig. 2D) in response to the largest amplitude perturbation. A hip abductor, GM activity also increased by an average of up to 15% during early to midstance (p’s < 0.024), but also by an average of up to 29% during terminal swing (p = 0.001; Fig. 3A), concurrent with significant increases in MH activity (p = 0.017; Fig. 3B). Also during terminal swing, EO activity increased by an average of up to 62% in response to perturbations (p = 0.049; Fig. 4A).

### Correlations between kinematic variability and EMG activity in the presence of perturbations

**Figure 5:**
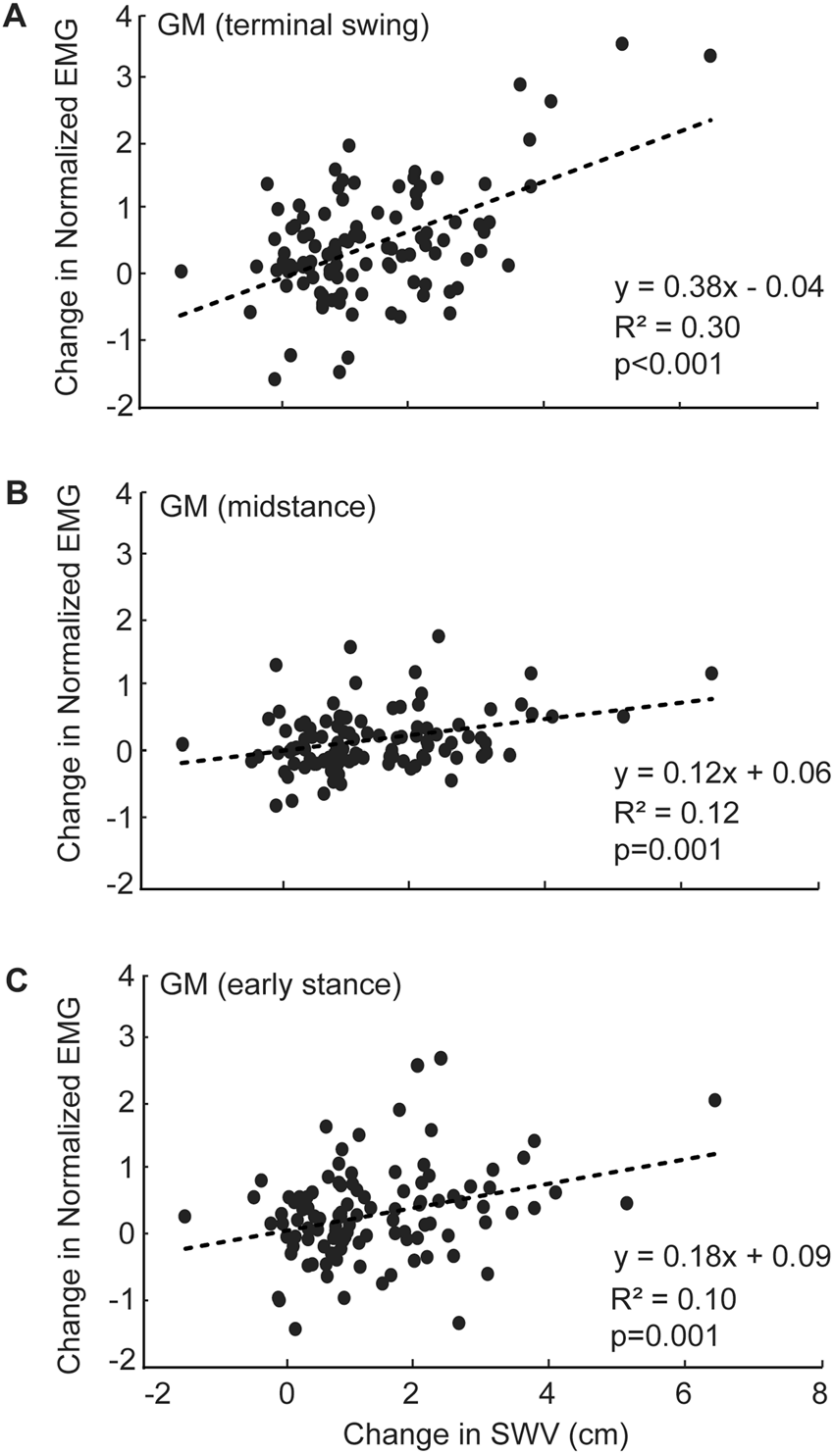
Correlations between changes in step width variability (SWV) and those in gluteus medius (GM) activity during (**A**) terminal swing, (**B**) midstance, and (**C**) early stance, pooled across walking speeds and perturbation amplitudes.

**Figure 6:**
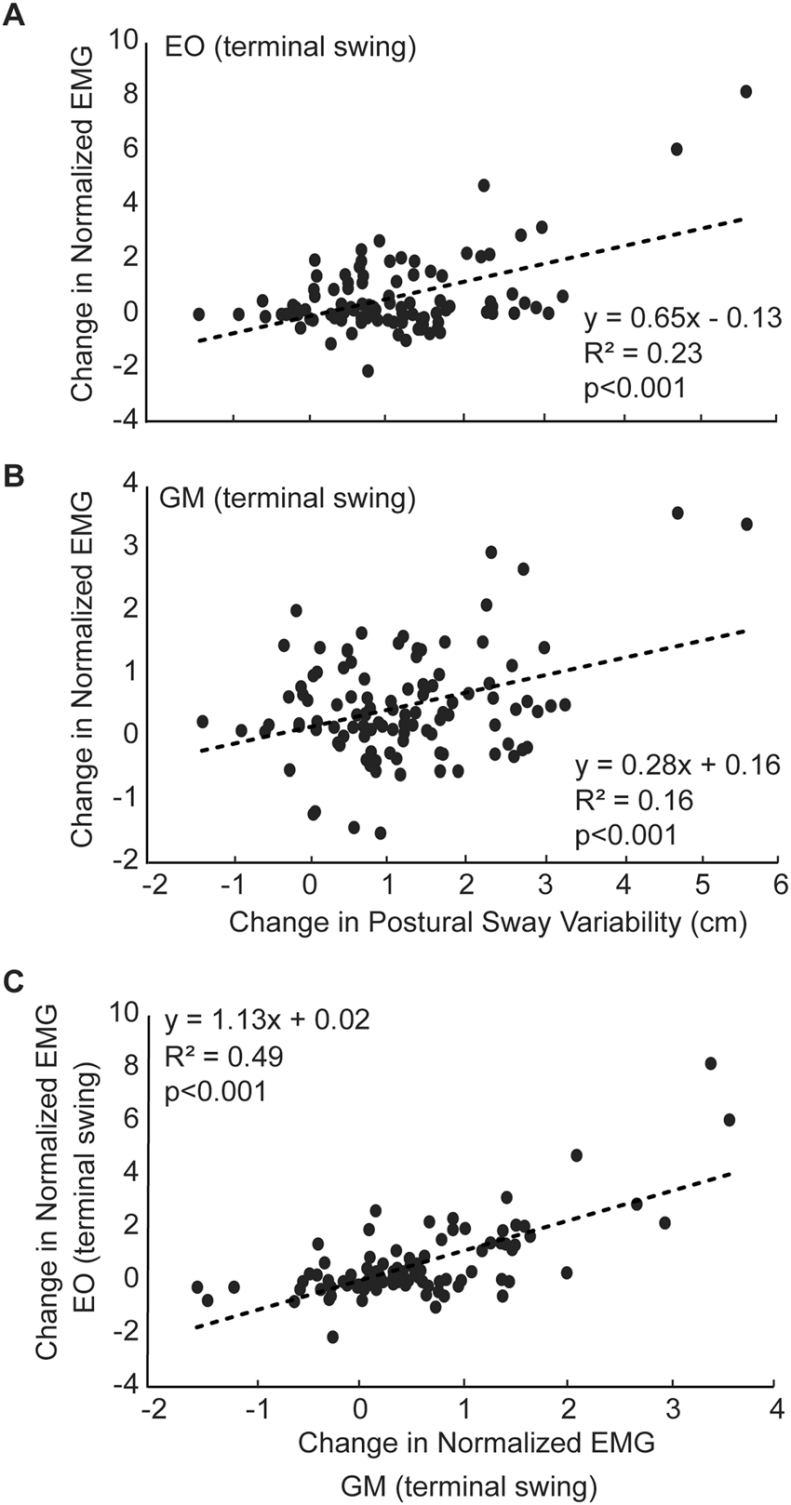
Correlations between changes in postural sway variability and those in (**A**) external oblique (EO) and (**B**) gluteus medius (GM) activity, both during terminal swing. (**C**) Correlations between concurrent changes in gluteus medius (GM) activity and those in external oblique (EO) activity during terminal swing. All correlations pooled across walking speeds and perturbation amplitudes.

We found moderate and significant positive correlations between perturbation-induced increases in SWV and those in GM activity during terminal swing (R^2^ = 0.30; Fig. 5A). We also found significant yet more modest positive correlations between these outcome measures during early to midstance (Fig. 5B,C). Perturbation-induced increases in postural sway variability correlated significantly with EO (R^2^ = 0.23; Fig. 6A) and GM (R^2^ = 0.16; Fig. 6B) activity during terminal swing. Finally, during the terminal swing phase, significant increases in EO activity in response to perturbations strongly and significantly correlated with concurrent increases in GM activity (R^2^ = 0.49; Fig. 6C).

### Effects of walking speed on response to perturbations

Walking faster increased SWV and all EMG amplitudes but decreased SW, SLV, and ML postural sway amplitude and variability (speed main effect, p’s < 0.029) (Fig. 1B,C). For example, during unperturbed walking, SWV increased by 12% and SLV decreased by 35% across the range of walking speeds tested (Fig. 1C). Post-hoc comparisons for the kinematic outcome measures revealed that walking faster muted the response to perturbations for SL, SLV, and SW (Fig. 1B,C). Based on significant speed by amplitude interactions, perturbation-induced effects were largest at 0.75 m/s for SW (p = 0.040; Fig. 1B), largest at 1.25 m/s for SWV (p = 0.013; Fig. 1C), but largest at 1.75 m/s for postural sway variability (p = 0.029; Fig. 1C). In addition, of the instrumented muscles, only the modest PL activity during midswing exhibited a significant speed by amplitude interaction, with larger perturbation effects at 1.75 m/s compared to the slower speeds (p = 0.01; Fig. 2D).

## Discussion

The scientific premise of this work was motivated by the potential for sensory perturbations to reveal fundamental insight into human walking balance control and translational insight into the onset and progression of balance deficits. We found that perturbations significantly increased step width, decreased step length, and elicited larger trunk sway compared to normal, unperturbed walking. However, perturbation-induced effects on the corresponding variabilities of these measurements, indicative of step to step kinematic adjustments, were much more profound. Kinematic variability is often used as a marker of balance control and greater variability is often ascribed to people at greater risk of falls^21, 22^. Thus, our primary purpose was to investigate the neuromuscular mechanisms governing observed increases in step to step kinematic adjustments in response to optical flow perturbations and their dependence on walking speed.

Compared to those during unperturbed walking, the activities of eight of nine instrumented trunk and leg muscles increased significantly in response to perturbations. But, these increases were phase-dependent in a manner that we interpret based on their functional relevance to walking balance control. First, as hypothesized, optical flow perturbations increased gluteus medius, external oblique, and erector spinae activities during ipsilateral leg swing. Growing evidence implicates gluteus medius activity during terminal swing in adjusting lateral foot placement to preserve walking balance^4, 23^. Indeed, a recent neuromechanical analysis combined EMG with perturbations of swing leg trajectories and concluded that gluteus medius activity during leg swing regulates the mediolateral translation of the trunk relative to the stance foot^4^. Here, we found that perturbation-induced increases in GM activity also progressed into the early to midstance phase. However, consistent with its purported role in governing lateral step placement, and also as hypothesized, changes in terminal swing GM activity exhibited a far stronger positive correlation with step to step adjustments in lateral foot placement.

In addition, we suspect that concurrent swing phase increases in erector spinae and external oblique activity serve to meet the demands of trunk lateral rotation and stability in the presence of perturbations. As hypothesized, changes in the magnitude of step to step adjustments in postural sway positively correlated with those of external oblique EMG in response to perturbations. We recently found evidence for a high degree of coordination between kinematic adjustments in posture and foot placement during walking that may be governed by neuromechanical mechanisms unique to perturbations of optical flow^10^. Specifically, we posit that optical flow perturbations elicit postural disturbances via visuomotor entrainment of head and trunk kinematics, unifying visual with somatosensory and vestibular feedback but subsequently eliciting step to step adjustments in lateral foot placement to preserve whole-body balance. Together, our current findings imply that trunk muscles such as the external oblique may actively orchestrate postural adjustments elicited by perturbations, while the gluteus medius may subsequently respond by orchestrating adjustments in lateral foot placement to preserve whole-body balance. In support of this coordination between posture and foot placement, we found that increases in GM activity and EO activity were not only simultaneous during the terminal swing phase, but were also significantly and strongly correlated (i.e., R^2^ = 0.49). Future neuromechanical modeling studies may provide the definitive insight needed to substantiate these empirical results.

We also hypothesized that perturbations would increase the coactivation of antagonistic leg muscles to improve joint stability, particularly with limb loading during the early stance phase. Antagonist leg muscle coactivation, often present in people with balance deficits, is considered a feedforward control strategy to increase leg joint stiffness and thereby aid in responding when balance is challenged^16, 24, 25^. In support of our hypothesis, we observed simultaneous perturbation-induced increases in TA, SOL, and MG activities, antagonistic muscles spanning the ankle, only during the early to midstance phase. In addition, we found concurrent increases in PL activity, a muscle also thought to play a role in ankle joint stability^26^. Together, these lower leg muscle responses suggest that the step to step kinematic adjustments used to accommodate balance perturbations during leg swing also increase the need for ankle joint stability during early to midstance. Our results are thus well aligned with the broader motor control literature, for example that from reaching tasks, during which antagonist muscle coactivation and limb stiffness increase in proportion to the magnitude of external force perturbations^14^.

Finally, some previous studies have found that walking slower improves metrics of dynamic stability, thought to characterize one’s resilience to perturbations, despite increasing kinematic variability^17^. Most of our outcome measures were speed-dependent and, in partial agreement with prior work^27–29^, we found that step length variability and postural sway variability increased with slower walking speed. However, interactions between walking speed and susceptibility to optical flow perturbations, when present, were more complex than anticipated. Of the speed by amplitude interactions examined across the kinematic measurements, only three emerged as statistically significant; one (i.e., step width) alluded to a larger response to perturbations at 0.75 m/s, one (i.e., step width variability) to those at 1.25 m/s, and one (i.e., postural sway variability) to those at 1.75 m/s. Compared to walking normally, subjects increased their step width in response to perturbations most at the slowest speed, 0.75 m/s. In contrast, step to step adjustments in step width (i.e., SWV) and postural sway variability increased least on average in response to perturbations applied at 0.75 m/s, a finding more consistent with the conceptual premise of Dingwell and Marin (2006). For example, perturbation-induced increases in postural sway variability were, on average, up to 81% larger at 1.75 m/s than at 0.75 m/s. Together, these findings suggest an increased dependence on, or at least utilization of, wider steps to withstand perturbations if walking balance is challenged at slower speeds, a general anticipatory strategy^30^ that may alleviate the need for more reactive step to step kinematic adjustments. Though, interestingly, we did not see nearly the same extent of speed by amplitude interactions for the EMG-based measurements. In fact, only perturbation-induced increases in peroneus longus activity were speed-dependent; these increases were significantly larger at the fast walking speed. This implies an increased demand for lateral ankle stability when balance is challenged at fast walking speeds. Ultimately, given the complex nature of interactions between walking speed and susceptibility to optical flow perturbations, we can only partially accept our second hypothesis that walking slower would improve one’s resilience to perturbations.

There are several limitations of this study that should be considered when interpreting our results. First, we only considered continuous, mediolateral optical flow perturbations that may not reflect kinematic and muscular adjustments, or correlations between them, when responding to a more discrete sense of imbalance. Given the continuous nature of the perturbations, we correlated subject-average increases in EMG activity with those in kinematic variability. However, there may be important and presumably complex step by step associations between muscle activity and kinematic variability that were not accounted for in this study, but could be perhaps through the use of more acute perturbations. In addition, we used complex perturbations that we presumed would be difficult for subjects to anticipate or predict. However, although unlikely, we cannot exclude the possibility that subjects learned to anticipate the perturbation dynamics. This is particularly relevant given our choice to analyze the second minute of walking data following the onset of perturbations, prior to which subjects could have exhibited some neuromuscular adaptation. While our intent was to allow some time for subjects to reach a steady state response, the reported perturbation-induced effects may be muted compared to those immediately following perturbation onset. In addition, although we studied a broad range of walking speeds, these speeds were fixed and constant on the treadmill. Subjects were thus unable to modulate their walking speed when responding to perturbations. Ultimately, it is not clear how these components of our experimental design may have influenced the results of this study.

## Conclusion

In conclusion, our findings point to specific muscular contributions to orchestrating the kinematic adjustments elicited by balance perturbations, in particular those of posture and lateral foot placement, in order to preserve whole-body balance. First, we found that perturbations increased EMG activity of the gluteus medius and postural control muscles in a coordinated manner during leg swing and the coactivation of antagonist lower leg muscles during limb loading in early stance. Second, we found that perturbation-induced increases in gluteus medius and postural control muscle activities positively correlated with step to step adjustments in lateral foot placement and postural sway, respectively. Finally, we found that interactions between walking speed and susceptibility to perturbations, when present, were more complex than anticipated. Ultimately, this study provides mechanistic insight into walking balance control and important reference values for the emergence of balance deficits due to aging or disease.
